# Effect of *Uncaria tomentosa* aqueous extract on the response to palmitate-induced lipotoxicity in cultured skeletal muscle cells

**DOI:** 10.1186/s12906-023-04204-4

**Published:** 2023-11-15

**Authors:** Bruna Leticia de Freitas-Marchi, Jeniffer Farias dos Santos, Gustavo Roncoli Reigado, Myrian Thiago Pruschinski Fernandes, Felipe Santiago Chambergo Alcalde, Carla Roberta de Oliveira Carvalho, Viviane Abreu Nunes

**Affiliations:** 1 Laboratory of Skin Physiology and Tissue Bioengineering, School of Arts, Sciences and Humanities, University of Sao Paulo (EACH-USP), São Paulo, SP Brazil; 2 Department of Physiology and Biophysics, Institute of Biomedical Sciences, University of São Paulo (USP), São Paulo, SP Brazil

**Keywords:** *Uncaria tomentosa*, Complementary therapy, Palmitate, Lipotoxicity, Reactive oxygen species, Oxidative stress, Insulin resistance, Type 2 diabetes mellitus, Skeletal muscle cells

## Abstract

**Background:**

Type 2 diabetes mellitus (T2DM) is frequently associated with dyslipidemia, which corresponds to the increase in the triglycerides and fatty acid concentrations in tissues, such as the skeletal muscle. Also, T2DM molecular mechanism involves increasing in reactive oxygen species (ROS) production and oxidative stress. The use of herbal medicines such as *Uncaria tomentosa* (Ut) has been proposed as an auxiliary treatment for patients with T2DM. In this study, it was evaluated the effect of Ut aqueous extract on cell viability and ROS production, in skeletal myoblasts from C2C12 lineage exposed to the free fatty acid palmitate (PA).

**Methods:**

Cells were incubated with PA in different concentrations ranging from 10 to 1000 μM, for 24 or 48 h, for cytotoxicity assay. Cell death, DNA fragmentation and ROS production assays were performed in cell cultures incubated with PA for 24 h, in the pre (preventive condition) or post treatment (therapeutic condition) with 250 μg/ml Ut aqueous extract, for 2 or 6 h. Cell death was evaluated by MTT method or flow cytometry. ROS generation was measured by fluorescence spectroscopy using the DCFDA probe.

**Results:**

Cell viability was reduced to approximately 44% after the incubation with PA for 24 h from the concentration of 500 µM. In the incubation of cells with 500 μM PA and Ut extract for 6 h, in both conditions (preventive or therapeutic), it was observed an increase of 27 and 70% in cell viability respectively, in comparison to the cultures incubated with only PA. Also, the incubation of cultures with 500 μM PA, for 24 h, increased 20-fold the ROS formation, while the treatment with Ut extract, for 6 h, both in the preventive or therapeutic conditions, promoted decrease of 21 and 55%, respectively.

**Conclusion:**

The Ut extract was efficient in promoting cell protection against PA lipotoxicity and ROS generation, potentially preventing oxidative stress in C2C12 skeletal muscle cells. Since T2DM molecular mechanism involves oxidative stress condition and it is often associated with dyslipidemia and fatty acid accumulation in muscle tissue, these results open perspectives for the use of Ut as an auxiliary strategy for T2DM management.

**Supplementary Information:**

The online version contains supplementary material available at 10.1186/s12906-023-04204-4.

## Introduction

Type 2 diabetes mellitus (T2DM) is a chronic noncommunicable disease with increasing prevalence in recent years [7, 53], resulting in low quality of life. It is estimated that in 2045, there will be an increase of 35% in cases worldwide, exceeding 690 million [10, 26, 57].

T2DM is considered a multifactorial syndrome characterized by chronic plasma hyperglycemia with disorders in the metabolism of carbohydrates, lipids and proteins, resulting from changes in insulin production, secretion and/or its mechanism of action. In addition, T2DM is often associated with dyslipidemia [13, 20, 51].

Diabetic dyslipidemia consists of increasing concentrations of triglycerides, which are composed of a glycerol associated with three fatty acids [19, 36]. Following lipolysis, free fatty acids are transported in the blood, mostly reversibly linked to albumin, and are mainly used by skeletal and cardiac muscle tissue as an energy source [3, 33].

In skeletal muscle, the accumulation of fatty acids such as palmitate (PA), which represents approximately 70 to 80% of the total plasma free fatty acids [25, 30], stimulates the production of proinflammatory cytokines and reactive oxygen species (ROS) [31, 35]. In addition, the increased PA concentration promotes mitochondrial dysfunction, which also leads to ROS production and oxidative stress [22, 35].

The involvement of ROS and cellular redox mechanismsin the regulation of several molecular processes has been shown [17, 22, 35]. In general, the antioxidant enzymes present in the tissues are sufficient to block the damage caused by physiological amounts of ROS. However, an imbalance between the formation of ROS and the ability of cells to remove these compounds results in oxidative stress [42, 56]. In particular, oxidative stress has been related to insulin resistance (IR) observed in T2DM [35], a condition in which cells do not respond adequately to circulating insulin levels. Although many molecular events can be related to IR, most are directly or indirectly associated with oxidative stress and inflammation.

Considering the molecular events involved in oxidative stress, the use of antioxidants combined with drugs that target lipid metabolism has been suggested for the treatment of obese individuals with T2DM [1, 4, 35].

Currently, it has been a challenge for health professionals and consumers to make decisions regarding safe combinations of drugs and medicinal plants due to a lack of strong clinical evidence related to plant compound interactions. In addition, little is known about the mechanisms involved in these processes [15, 55].

Most conventional antidiabetic drugs are described to cause side effects in long-term use, and options from natural products, especially herbal medicines, are present in the scientific literature and cited as an auxiliary choice to be used overlapping with traditional medicines [46, 47].

For many years, knowledge about plants has demonstrated that they are a rich source of pharmacologically active phytochemicals and therapeutic moieties [27]. They have been used as an efficient complementary therapy for several pathological processes [37, 45].

In 2013, the WHO (World Health Organization) published the “WHO traditional medicine strategy: 2014–2023”, which aims to support the practice of complementary and alternative medicine to promote public health, including phytotherapy, which is the use of medicinal plants for a healthy application. The plan aims to promote the safety, efficacy, and quality of complementary and alternative medicine by expanding the knowledge base and providing guidance under regulatory and quality assurance standards [52]. In 2016, the American National Center for Complementary and Integrative Health (NCCIH) published a strategic plan to explore the science of complementary and integrative health, including phytotherapy [38]. In this context, several plants of the Brazilian flora have been explored, focusing on therapies for the treatment of different diseases.

*Uncaria tomentosa* (Ut), popularly known as “cat’s claw”, is a plant species of the Rubiaceae family recognized as an herbal medicine that appears in the National List of Essential Medicines (RENAME) [8] for use in capsules, gels or teas.

It has already been shown in in vitro tests that Ut bark aqueous extract presents antioxidant activity [29, 39]. Other studies have also shown the presence of antioxidant compounds such as cinchonain, a molecule belonging to the proanthocyanidin class [2, 23, 50]. In this sense, using a cell culture model, Bors et al. [6] confirmed the antioxidant effect of Ut leaf aqueous preparations on human erythrocytes against oxidative stress. It has also been shown that Ut aqueous extract prevents lipid peroxidation in phagocytic cells and the consequent damage to the cell membrane and DNA [6, 21].

We have shown that the viability of muscle cells was significantly reduced in cultures incubated with 500 μM PA for 24 or 48 h [18]. Considering the convergence of multiple processes in T2DM, such as cell death, oxidative stress, dyslipidemia and fatty acid accumulation in skeletal muscle cells, we have shown here the effect of Ut aqueous extract on palmitate-induced cell death and oxidative stress in myoblasts.

## Material and methods

All methods were carried out in accordance with relevant guidelines and regulations.

### Cell cultures

C2C12 skeletal muscle cells, a cell lineage derived from mice (ATCC—American Type Culture Collection, CRL—1772) were acquired from Rio de Janeiro Cell Bank (BCRJ). Cells were cultured in Dulbecco’s modified Eagle’s medium (DMEM) containing 4 mM glucose, 10% fetal bovine serum (FBS) and the antibiotics 10,000 IU/ml penicillin and 10 mg/ml /streptomycin, under 5% CO_2_ at 37 °C, until the culture reach 80% of confluence. For the different experiments, cells were transferred to 6- or 96-well plates.

### Ut aqueous extract and palmitate solutions preparation

The powder of *Uncaria tomentosa* crude extract (Reference number 100001372, code 2470), from the plant root bark, was donated by Herbarium Botanic Laboratory (herbarium.com.br, Curitiba, PR, Brazil). A powdered fraction was used to prepare the aqueous extract. The solution was prepared weekly using 100 mg of the extract in 1 ml of distilled water (dH_2_O), which was kept at 37 °C for 24 h according to Bors et al. [6]. After this period, the solution was homogenized and centrifuged for 5 min, at 400 × g. The supernatant was recovered and filtered (0,22 μm) to be used in the experiments. The chromatogram of the crude extract, provided by the supplier (Supplementary Information S1), confirms the presence of some compounds typically used as confirmatory profiles to the species *tomentosa*, such as *mitraphylline* or *isomitraphylline*.

The PA stock solution was prepared at the concentration of 0,1 M in absolute ethanol. At the moment of incubating with cultures, the stock solution was diluted to the final concentration, as described in each experiment, and 2% bovine serum albumin (BSA) was added to conjugate the fatty acid to the protein.

### Cell treatments

Cells were plated at 2 × 10^5^ or 1 × 10^4^ cells/ml density, in 6 or 96-well plates, respectively, and incubated with the PA solution in different concentrations (0, 10, 50, 100, 250, 500, 750 and 1000 μM), for 24 or 48 h, for cytotoxicity assays. The effect of Ut extract, in different concentrations (0, 10, 50, 100, 250, 500, 750 and 1000 μg/ml), was preliminarily evaluated on the incubation for 24 h with skeletal muscle cell viability. The prevention of cell death, DNA fragmentation and ROS production induced by the incubation of cells with PA, for 24 h, was studied under the preventive or therapeutic treatments, for 2 or 6 h, with the 250 μg/ml Ut aqueous extract, as indicated in each experiment.

**Fig. 1:**
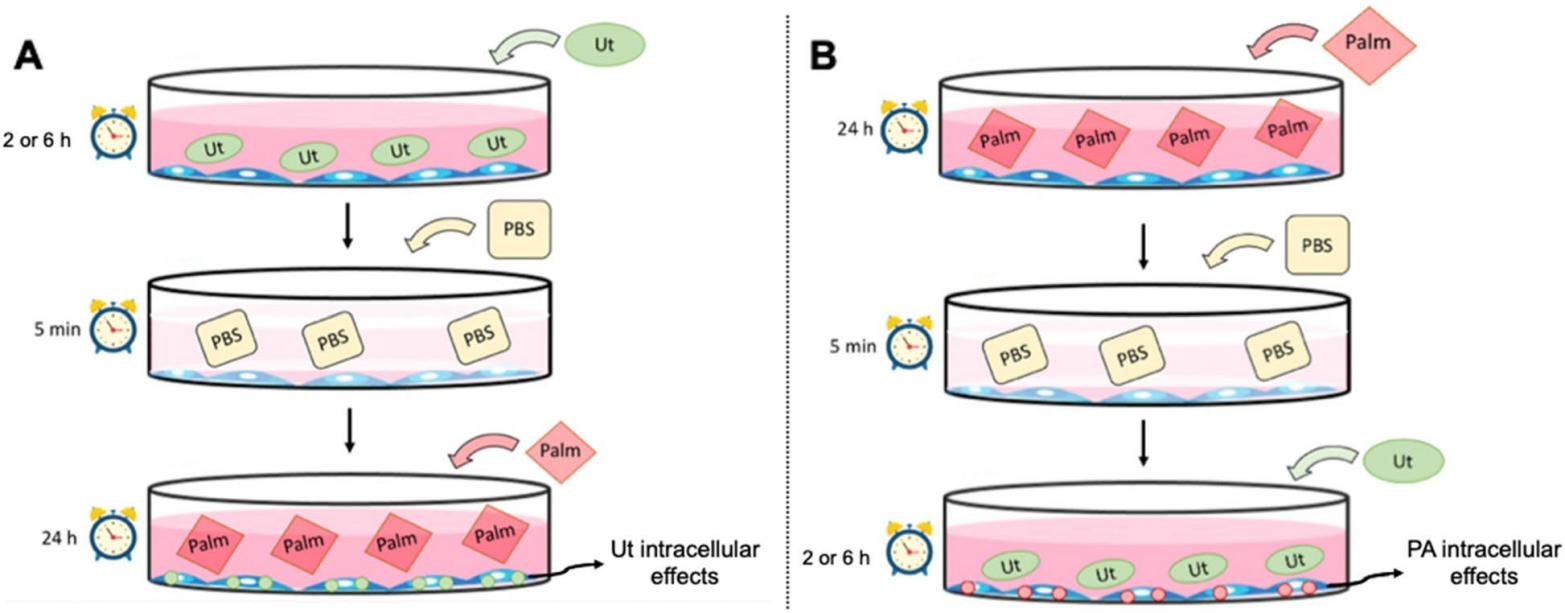
Schematic experimental approaches. **A** Cells were treated, for 2 or 6 h, with *Uncaria tomentosa* (Ut) aqueous extract; after these periods, cultures were washed with PBS and incubated with PA solution in different concentrations, for 24 h (preventive condition); **B** Cells were incubated with PA in different concentrations, for 24 h; after, cultures were washed with PBS and, then, Ut aqueous extract was added, for 2 or 6 h (therapeutic condition) and analyzed after these periods

The Fig. 1 summarizes the experimental approaches to study Ut extract effect on PA-induced muscle cell death, DNA fragmentation and ROS generation: A) Cultures were treated, for 2 or 6 h, with 250 μg/ml Ut aqueous extract (preventive condition), then they were washed with phosphate buffered saline (PBS) before the incubation with PA in different concentrations, and analyzed after 24 h; B) Cultures were first incubated with PA in different concentrations for 24 h, then they were washed with PBS and subsequently treated with 250 μg/ml Ut aqueous extract, for 2 or 6 h (therapeutic condition).

### Cell viability analysis by MTT method

The 3-(4,5-dimethylazol-2-yl)-2,5-diphenyltetrazolium bromide (MTT) stock solution was prepared at 5 mg/ml in PBS. After the incubation with PA for 24 h, in the absence or presence of pretreatment with Ut aqueous extract (preventive condition), 5 μl of MTT solution were added to the cultures in 96-well microplate and the cells were cultured for 4 h, at 37 °C. After this period, the medium was removed and 100 μl of dimethyl sulfoxide (DMSO) was added to each well to dissolve formazan crystals. Cell viability was determined by the absorbance at 595 nm in the BioTek Synergy HT microplate reader. Four individual experiments were performed in triplicates and the results were analyzed with Gen5 software. The absorbance was converted into the percentage (%) of viable cells in relation to the total, as following:

$$\mathrm{Total\ \%}=\left(\left[\frac{\mathrm{Abs}\left(\mathrm{sample}\right)}{\mathrm{Abs}\left(\mathrm{control}\right)}\right]\times 100\right)-100$$

The degree of cytotoxicity was determined using the classification by Oliveira [41]. Samples incubated with PA were compared to control (cells cultured in the presence of 0,5% ethanol).

### Cell viability and DNA fragmentation analysis by flow cytometry

For these assays, incubation with PA was performed only for 24 h, in the absence or presence of treatment with Ut aqueous extract (preventive condition). Cells were, then, collected with 0.25% trypsin. For each sample, two tubes were used to separate the pellets and evaluate cell viability or DNA fragmentation. The cell suspension was centrifuged for 5 min at 400 × g. To assess cell viability, the pellet was resuspended in 200 μl of PBS, and 5 μl of 1 mg/ml propidium iodide (PI) was added to the cell suspension. For DNA fragmentation analysis, the pellet was resuspended in 300 μl of PBS containing 0.1% Triton® X-100 and 20 μg/ml PI. Cells were evaluated on Guava EasyCyte 8HT flow cytometer using the InCyte® software. Three individual experiments were performed in triplicates and 10.000 events were acquired per sample. The results were expressed as a percentage of the total number of cells in each sample.

### Intracellular ROS production

After incubation of cell cultures with 100 or 500 μM PA, in the absence or presence of pretreatment with Ut aqueous extract (preventive condition), the ROS production was quantified using the fluorescent marker 2',7'-dichlorofluorescein diacetate (DCFDA) using the commercial kit DCFDA/HeDCFDA d6883 (Sigma-Aldrich, St. Louis, USA). The medium was removed from the plates and two washings with PBS were performed. Then, 10 μM DCFDA diluted in DMEM without phenol red was added and the incubation continued for 30 min at 37 °C. Cultures were washed twice with PBS and the ROS presence was evaluated at λ_excitation_ = 485 nm and λ_emission_ = 528 nm in the BioTek Synergy HT microplate reader. Three individual experiments were performed in triplicates and the results were analyzed using the Gen5 software (version 1.0.14, BioTek). The fluorescence values were expressed in Fluorescence Arbitrary Units (FAU) and converted into percentage, considering the number of viable cells in each sample, in relation to the control (cells cultured in the presence of 0,5% ethanol).

### Statistical analysis

The results were expressed as the mean ± standard error (SE) and the differences were analyzed by two-way ANOVA, followed by the Bonferroni post-test, using the GraphPad Prism software (version 8). The differences were considered significant at *p* < 0.05.

## Results

### PA and Ut extract cytotoxicity analysis

**Fig. 2:**
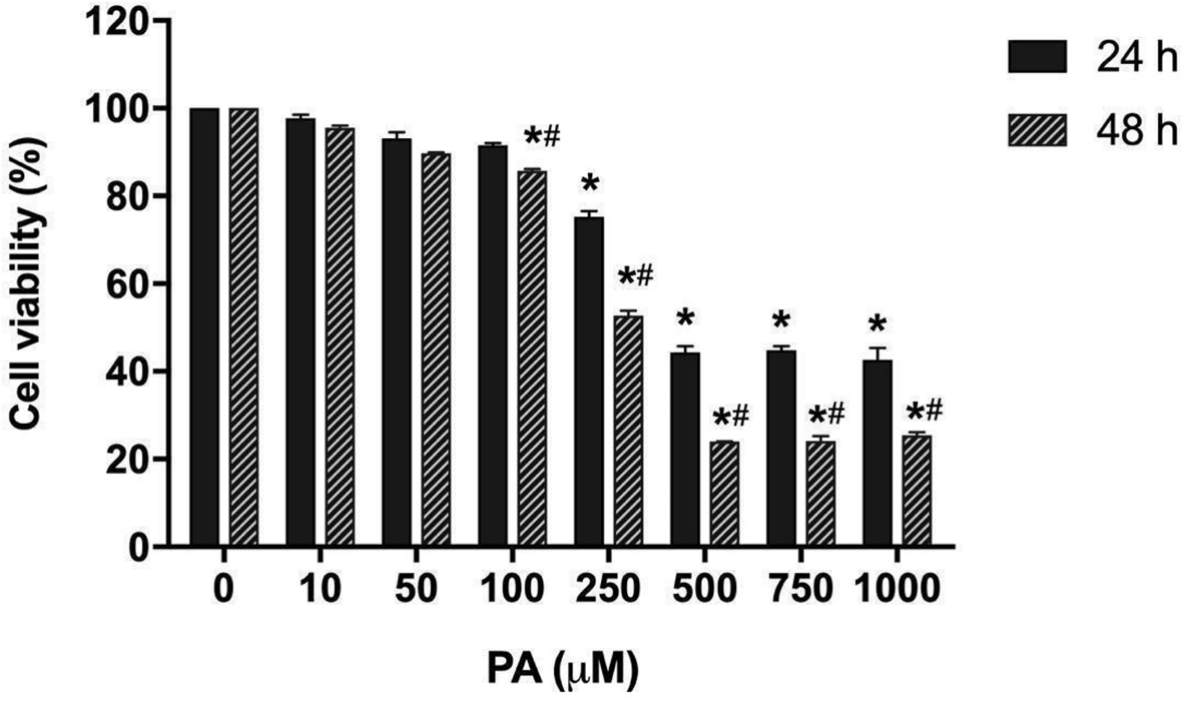
Effect of palmitate (PA) in the viability of C2C12 myoblasts. Cells were incubated, for 24 or 48 h, with PA (10 to 1000 μM) and viability was evaluated by the MTT method. The data are presented as the mean ± SE of four individual experiments in triplicates. The values are expressed as the percentage in relation to the control. * Indicates difference compared to the control without the PA; # indicates difference between the two periods of time (*p* < 0.05)

Cell viability was evaluated in cultures exposed to PA in different concentrations. It was observed a reduction in cell viability to approximately 44 and 26% in the incubation with 500, 750 and 1000 μM PA, for 24 or 48 h, respectively, compared to the control (Fig. 2). However, this free fatty acid was not considered toxic at the concentrations of 10, 50 and 100 μM. Considering these results, the concentrations of 100 and 500 μM, under the period of 24 h, were used as non-cytotoxic and cytotoxic, respectively, in the following experiments.

**Fig. 3:**
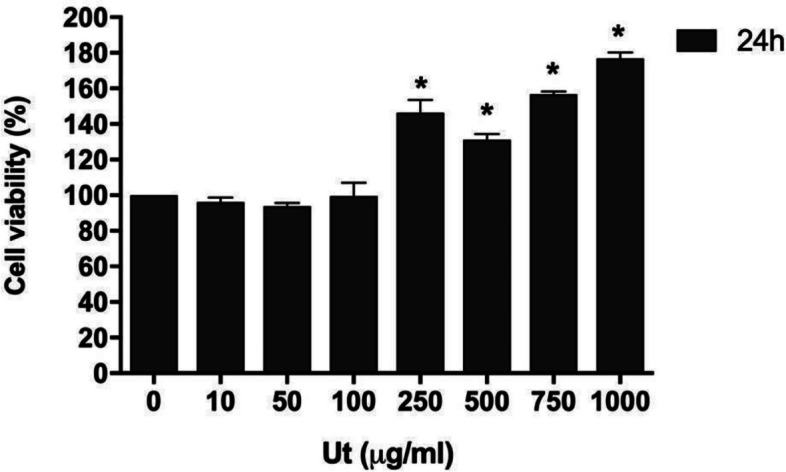
Effect of the *Uncaria tomentosa* (Ut) aqueous extract on the viability of C2C12 myoblasts. Cells were incubated, for 24 h, with the Ut aqueous extract (10, 50, 100, 250, 500, 750 and 1000 μg/ml) and evaluated by the MTT method. The data are presented as the mean ± SE of four individual experiments in triplicates. The values are expressed as the percentage in relation to the control. * Indicates difference in comparison to the control without the Ut extract (*p* < 0.05)

The incubation of cells with Ut aqueous extract starting from 250 μg/ml resulted in significant increase in cell viability (from 30 to 70%) (Fig. 3). Since the concentration of 250 μg/ml was the lowest capable of promoting increase in cell viability in 24 h, this was the one chosen for further experiments.

### Ut extract effect on PA-induced cytotoxicity

**Fig. 4:**
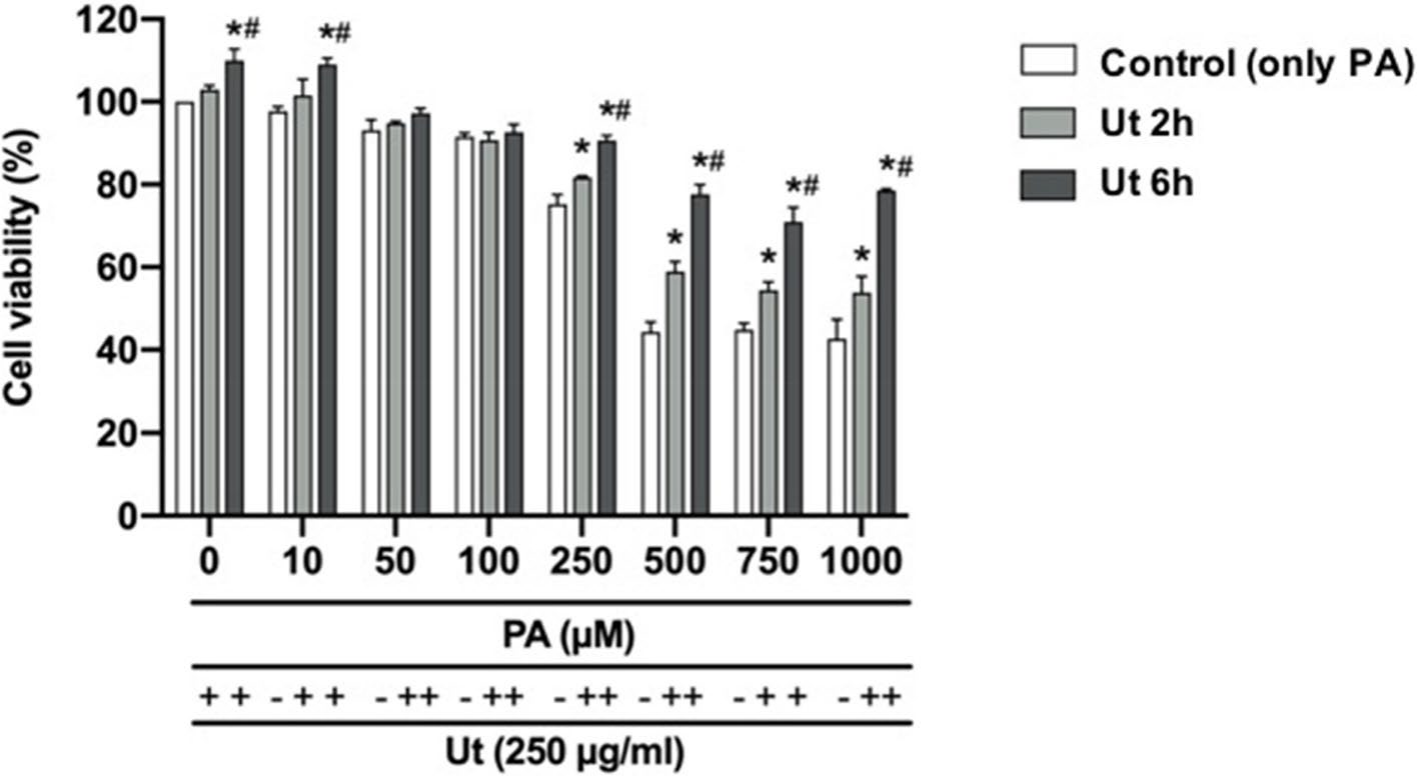
Effect of the Ut aqueous extract against the PA cytotoxicity in C2C12 myoblasts. Cultures were treated with the 250 μg/ml Ut extract in the preventive condition, for 2 or 6 h, and incubated with PA in different concentrations, for 24 h. Cell viability was assessed using the MTT method. Data are presented as the mean ± SE of four individual experiments in triplicates and the values are expressed as the percentage, in relation to the control with only PA. * Indicates difference in comparison to the control of each group; # indicates the difference between the treatment with Ut for 2 or 6 h (*p* < 0.05)

Cell viability in cultures treated with 250 μg/ml Ut extract in the preventive condition, for 2 or 6 h, and incubated with PA in concentrations above 500 μM, was increased between 30 and 70% respectively, in comparison to control that corresponds to cultures incubated with only PA (Fig. 4).

The pretreatment of cells with Ut extract for 6 h resulted in 20% increase in cell viability, in cultures incubated with PA at concentrations higher than 500 µM, compared to the period of 2 h, which suggests a time dependent effect.

### Cell viability and DNA fragmentation analysis by flow cytometry

**Fig. 5:**
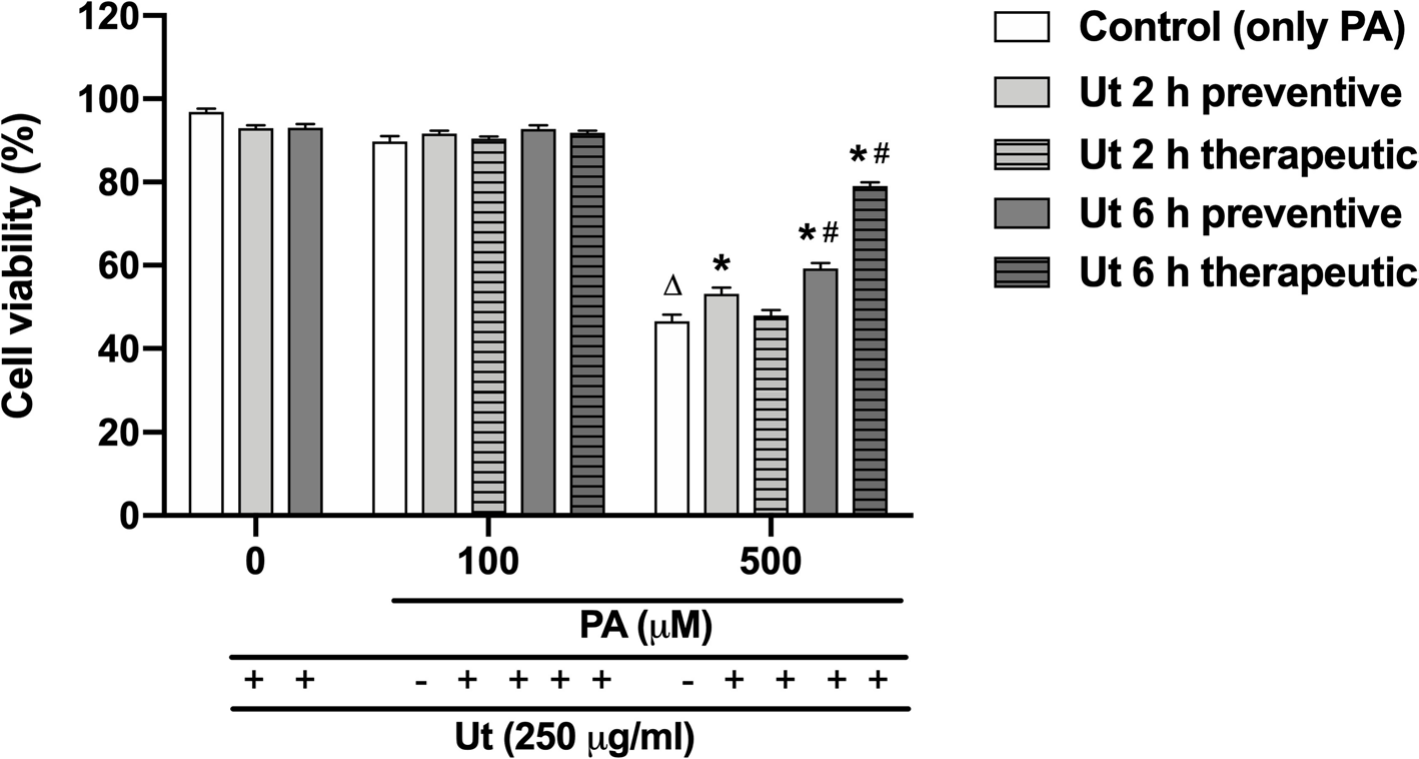
Effect of the Ut aqueous extract against the PA cytotoxicity in C2C12 myoblasts. Cells were treated with the 250 μg/ml Ut aqueous extract in preventive or therapeutic conditions, for 2 or 6 h, and incubated with 100 or 500 μM PA, for 24 h. Cell viability was assessed by flow cytometry using propidium iodide. 10.000 events were acquired per sample. The data are presented as the mean ± SE of three individual experiments in duplicates and the values are expressed as the percentage in relation to the control. * Indicates difference compared to the control with only PA; # indicates difference between treatments with Ut extract for 2 or 6 h; ∆ indicates difference in comparison to cultures incubated with only ethanol (*p* < 0.05)

The treatment of cells with only 250 μg/ml Ut extract for 2 or 6 h did not result in significant alterations in cell death, compared to the control with no Ut (Fig. 5). However, when the cultures were treated with the extract for 2 h in the preventive condition, and subsequently exposed to 500 μM PA, there was an increase of 12% in cell viability compared to the incubation with only the free fatty acid. Also, when the cells were incubated with 500 μM PA and treated with Ut extract for 6 h, in both conditions (preventive or therapeutic), it was observed an increase of 27 and 70% in cell viability, respectively, in relation to the cultures incubated with only PA.

**Fig. 6:**
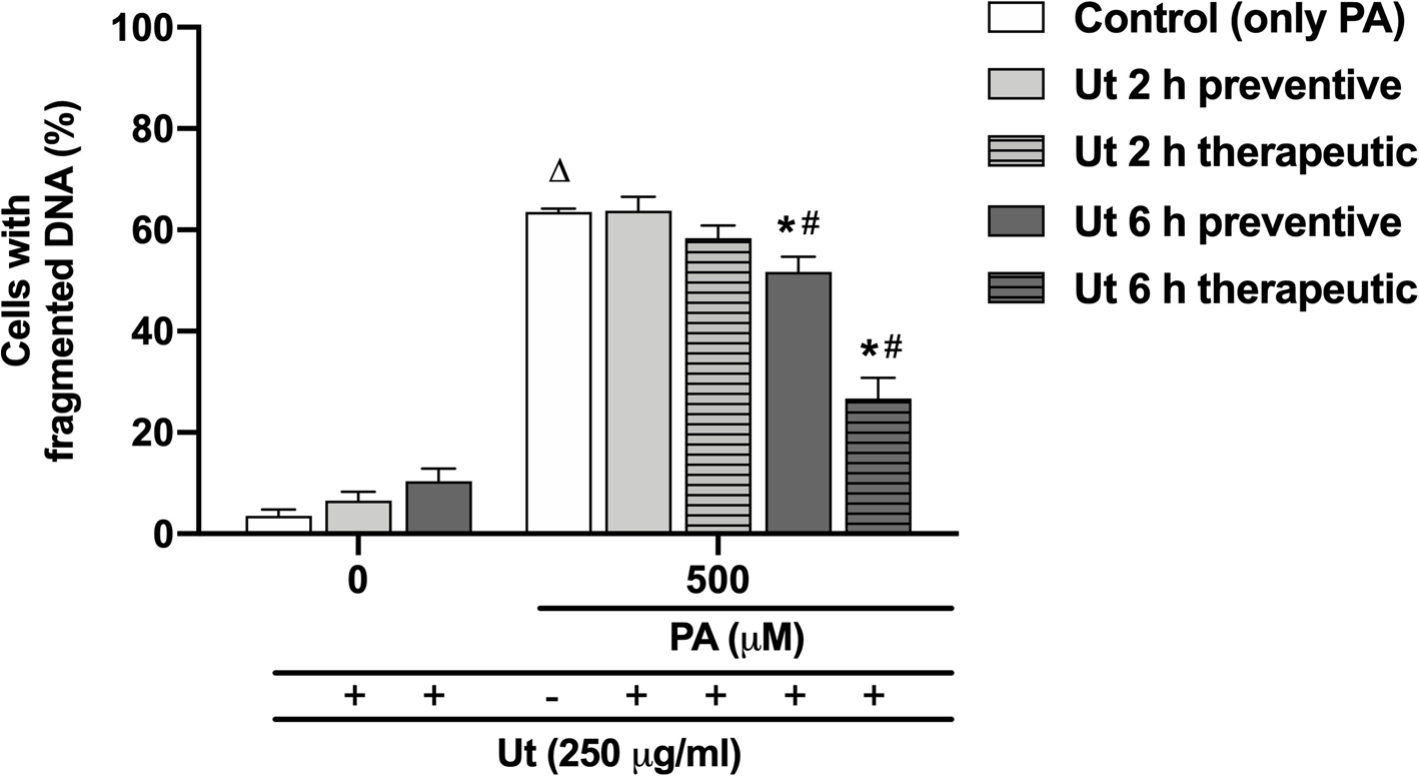
Effect of the Ut aqueous extract on palmitate-induced DNA fragmentation in C2C12 myoblasts. The cells were treated with the 250 μg/ml Ut extract in the preventive or therapeutic conditions, for 2 or 6 h, and incubated with 100 or 500 μM PA, for 24 h. 10.000 events were acquired per sample. The data are presented as the mean ± SE of three individual experiments in duplicates and the values are expressed as the percentage in relation to the control, according to the number of viable cells in each sample. * Indicates difference in relation to the control with only PA; # indicates the difference between both preventive or therapeutic treatments with Ut for 2 or 6 h; ∆ indicates difference in relation to the cultures incubated with only ethanol (*p* < 0.05)

In order to study molecular events related to palmitate-induced cell death, the DNA fragmentation was evaluated, as an indicative of apoptosis occurrence. In the absence of cytotoxic stimulus, the Ut aqueous extract had no significant effect on DNA fragmentation compared to the control (Fig. 6). In cultures incubated with 500 μM PA, the percentage of cells with fragmented DNA was 60%, which is statistically different from the control. Furthermore, in cultures incubated with 500 μM PA and treated with the Ut aqueous extract, for 6 h, in the preventive or therapeutic conditions, it was observed a reduction of 16% and 57%, respectively, in the number of cells with fragmented DNA, compared to cultures incubated with only PA.

### Ut extract effect on ROS intracellular production in PA-induced cell death

**Fig. 7:**
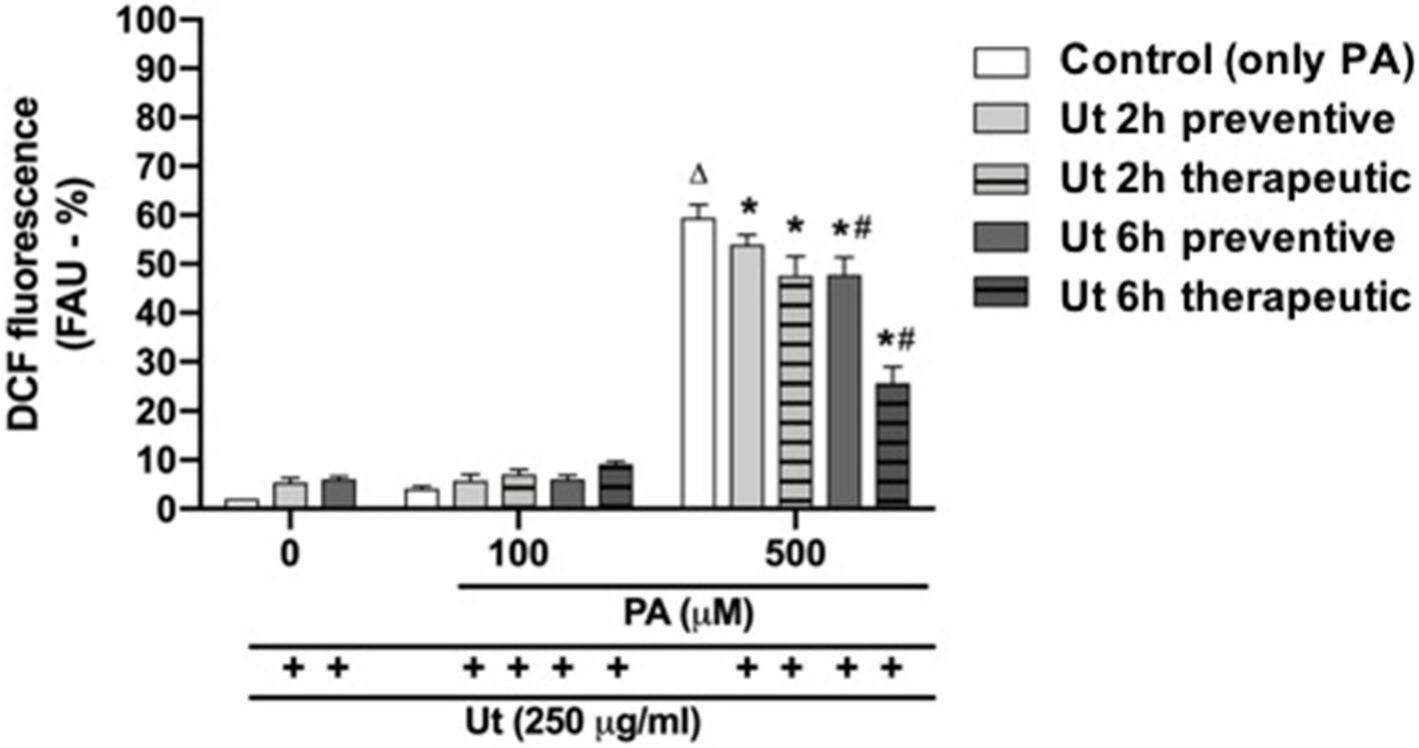
Quantification of ROS production in C2C12 myoblasts incubated with PA, in the absence or presence of treatment with Ut extract. The samples were evaluated by fluorescence spectroscopy, at λ_excitation_ = 485 nm and λ_emission_ = 528 nm. The ROS formation was measured in fluorescence arbitrary units (FAU) and expressed as the percentage considering the number of viable cells in each sample, in relation to the control. The data are presented as the mean ± SE of three individual experiments in triplicates. * Indicates difference in relation to the control with only PA; # indicates difference between treatments with Ut extract for 2 or 6 h; ∆ indicates difference in relation to cultures incubated with only ethanol (*p* < 0.05)

The incubation of cultures with 100 μM PA, in the absence or presence of Ut aqueous extract treatment in the preventive condition, did not increase the percentage of ROS formation, in relation to the cultures incubated with only PA (Fig. 7). However, the incubation of cultures with 500 μM PA, for 24 h, increased 20-fold the ROS formation compared to the control, indicating its involvement in ROS generation and, potentially, in oxidative stress.

Interestingly, the treatment with Ut aqueous extract, for 2 h, in the preventive or therapeutic conditions, resulted in a significant reduction of 12 and 20%, respectively, in ROS production when compared to cultures treated with only 500 μM PA, while the treatment with Ut extract, for 6 h, both in the preventive or therapeutic conditions, promoted decrease of 21 and 55%, respectively, in the ROS formation, compared to control with only the free fatty acid.

## Discussion

Considering the convergence of multiple processes, such as cell death, oxidative stress, inflammation, dyslipidemia, IR and DM2, and that skeletal muscle tissue is an important target for the high content of plasma triglycerides and free fatty acids, several experimental models have been developed to evaluate PA lipotoxicity using rat femoral and mesenteric arteries or mouse-isolated skeletal muscle cells [34, 44, 49].

Here, we developed a simplified in vitro model to evaluate the cytotoxic effect of PA on skeletal muscle cells of the C2C12 lineage. PA cytotoxicity was studied at concentrations that can be achieved during overnight fasting (100 μM), prolonged fasting (250 μM) or in uncompensated T2DM (1000 μM) [11, 24]. We have shown that PA at a concentration of 500 μM was cytotoxic to myoblasts when incubated for 24 or 48 h [18]. Similarly, Filippello et al. [16] demonstrated that the incubation ofmurine intestinal cells with PA at concentrations higher than 500 µM for 24 or 48 h resulted in a reduction in cell viability of 40 and 60%, respectively, in a time- and concentration-dependent manner. In addition, the incubation of cultures with PA resulted in a significant rise in the number of cells with fragmented DNA, which is an indication of apoptosis occurrence [40, 43]. It was also shown that the incubation of cultures with 500 μM PA for 24 h increased ROS formation by 20-fold compared to the control, confirming its involvement in ROS generation and, potentially, in oxidative stress.

On the other hand, the treatment of cultures with 250 μg/ml Ut aqueous extract promoted an increase of 50% in cell viability compared to the control. Comparable results were obtained by Ciani et al. [12] using immortalized skin cells, which showed that incubation with Ut aqueous extract at concentrations from 250 to 1000 μg/ml for 24 h promoted an increase in cell viability between 40 and 60%.

We have tried two different approaches (preventive and therapeutic conditions) to evaluate the Ut aqueous extract effect on myoblast viability. In this sense, when the cultures were pretreated with Ut aqueous extract for 2 or 6 h, followed by incubation with PA, an increase in cell viability by 55%, on average, was verified when compared to incubation with only free fatty acids [18].

To evaluate whether the increase in myoblast viability occurred due to the presence of Ut extract in the culture medium during incubation with PA or if compounds present in the Ut extract might participate in the cellular response against PA cytotoxicity, cultures were washed with PBS prior to incubation with PA. Under this preventive condition, an increase of 45% in cell viability was observed on average. This result suggests that some compounds present in the herbal extract might have protected the cells against PA lipotoxicity, such as peroxidation of cell membrane lipids and/or DNA damage, which are associated with cell death. Additionally, according to Ying et al. [54], compounds in Ut extract might enter the cells, directly protecting cellular organelles and/or are capable of activating intracellular antioxidant defense pathways. Similar findings were obtained by Duchnowicz et al. [14], who showed that compounds from Ut bark extract not only penetrate deeper layers of the cell membranes of erythrocytes but are also transferred inside the cells. Such effects of Ut bark extract compounds were also described by Tarahovsky et al. [48], which suggested that secondary metabolites are able to penetrate the hydrophobic site of the lipid bilayer, particularly into the compartments known as lipid rafts, and increase the antioxidative potential, facilitating the protection of membrane lipids against oxidation.

Based on this evidence, we also evaluated the effect of Ut extract on the recovery of cell viability following the incubation of cultures with 500 μM PA in the treatment called therapeutic conditions [28]. Under this condition, an increase of up to 70% in cell viability was observed, indicating that 250 µg/ml Ut extract attenuated PA-induced lipotoxicity in myoblasts. In particular, treatment with Ut aqueous extract for 6 h was more effective for cell viability recovery, indicating a time-dependent effect and suggesting its use even after cells have been damaged by free fatty acid lipotoxicity.

Treatment with Ut aqueous extract for 2 or 6 h, under both preventive and therapeutic conditions, resulted in a reduction in ROS generation compared to that in cultures incubated with only 500 μM PA. Treatment for 6 h appeared to be more efficient in comparison to the shorter period, as also observed for cell viability results. Interestingly, treatment with Ut extract for 2 or 6 h resulted in a small but not significant increase in ROS production compared to the control. This dual response could be due to the variety of secondary metabolites present in Ut aqueous extract, as reported by Heitzman et al. [23] and Ciani et al. [12], which could also be related to the small percentage of cells with fragmented DNA verified in the treatments with only Ut extract. According to Calabrese & Mattson [9], this biphasic response of cells or organisms may represent an adaptive response to different stimuli, known as the hormesis effect. Indeed, we showed that both preventive and therapeutic conditions with the Ut aqueous extract were efficient for cell viability recovery, promoting a reduction in the percentage of cells with fragmented DNA and in the production of ROS in cells exposed to 500 μM PA.

The effect of Ut on skeletal muscle cells exposed to PA has been attributed to its antioxidant content, which can neutralize ROS and other free radicals, preventing oxidative damage to membrane lipids and DNA [5, 23, 32]. Specifically, Abouelela et al. [1] investigated the antioxidant effect of the alkaloid *cinchonain*, isolated from the aqueous extract of the *Ceiba pentandra* plant, and tested it in adult rats with renal deficiency. They found in assays for ROS quantification and immunohistochemistry analysis that *cinchonain* was able to increase hepatocyte viability, which was correlated with its antioxidant potential.

In summary, we have shown here that Ut extract was efficient in promoting cell protection against PA lipotoxicity and ROS generation, potentially preventing oxidative stress in muscle cells of the C2C12 lineage. These results might contribute to the understanding of the molecular mechanism involved in Ut aqueous extract action on PA-induced lipotoxicity in skeletal muscle tissue, opening perspectives for further studies based on redox state reestablishment using herbal-derived formulations for T2DM auxiliary treatment.

## Conclusions

Since T2DM pathogenesis and molecular mechanism involves oxidative stress and is often associated with the increase of free fatty acid concentration in the muscle tissue, we have shown that the viability of myoblasts incubated with PA at concentrations above 500 μM, for 24 or 48 h, was significantly reduced, confirming the lipotoxic effect of this free fatty acid.

The incubation of cultures with Ut aqueous extract at the concentration of 250 μg/ml, for 2 or 6 h, in both preventive or therapeutic conditions, resulted in increase in cell viability, decrease in DNA fragmentation and reduction in ROS production in muscle cells exposed to 500 μM PA, which is considered a cytotoxic concentration.

Finally, these results open perspectives for testing Ut extract and its isolated compounds in in silico and in vivo approaches in order to better characterize the mechanisms involved on the response to ROS production, oxidative stress, lipotoxicity and cell death, aiming for future use as an auxiliary strategy for T2DM management.

### Supplementary Information

**Additional file 1: SI 1.** Chromatogram of *Uncaria* *tomentosa* crude extract used in the experiments, showing most of the alkaloids such as uncarine D, uncarine F, isomitraphylline, uncarine C, uncarine E, as well as mitraphylline (5.97%), which is a major component of the oxindole alkaloids. The crude extract compounds presented until one hundred milli-Absorbance Units (mAU), measured by high-performance liquid chromatography (HPLC).

## Data Availability

Previously reported data used to support this study are available at https://doi.org/10.22533/at.ed.40420240616. This prior study of our group is cited as the reference Freitas et al. [18].
