# Supplementary material for: Effect of Uncaria tomentosa aqueous extract on the response to palmitate-induced lipotoxicity in cultured skeletal muscle cells
Source: BMC Complement Med Ther. 2023 Nov 15;23:412. doi: 10.1186/s12906-023-04204-4 (PMC10647034; doi:10.1186/s12906-023-04204-4)
Supplement: Supplementary file 1 — Additional file 1: SI 1. Chromatogram of Uncaria tomentosa crude extract used in the experiments, showing most of the alkaloids such as uncarine D, uncarine F, isomitraphylline, uncarine C, uncarine E, as well as mitraphylline (5.97%), which is a major component of the oxindole alkaloids. The crude extract compounds presented until one hundred milli-Absorbance Units (mAU), measured by high-performance liquid chromatography (HPLC). [file 12906_2023_4204_MOESM1_ESM.docx]

**SUPPLEMENTARY INFORMATION**


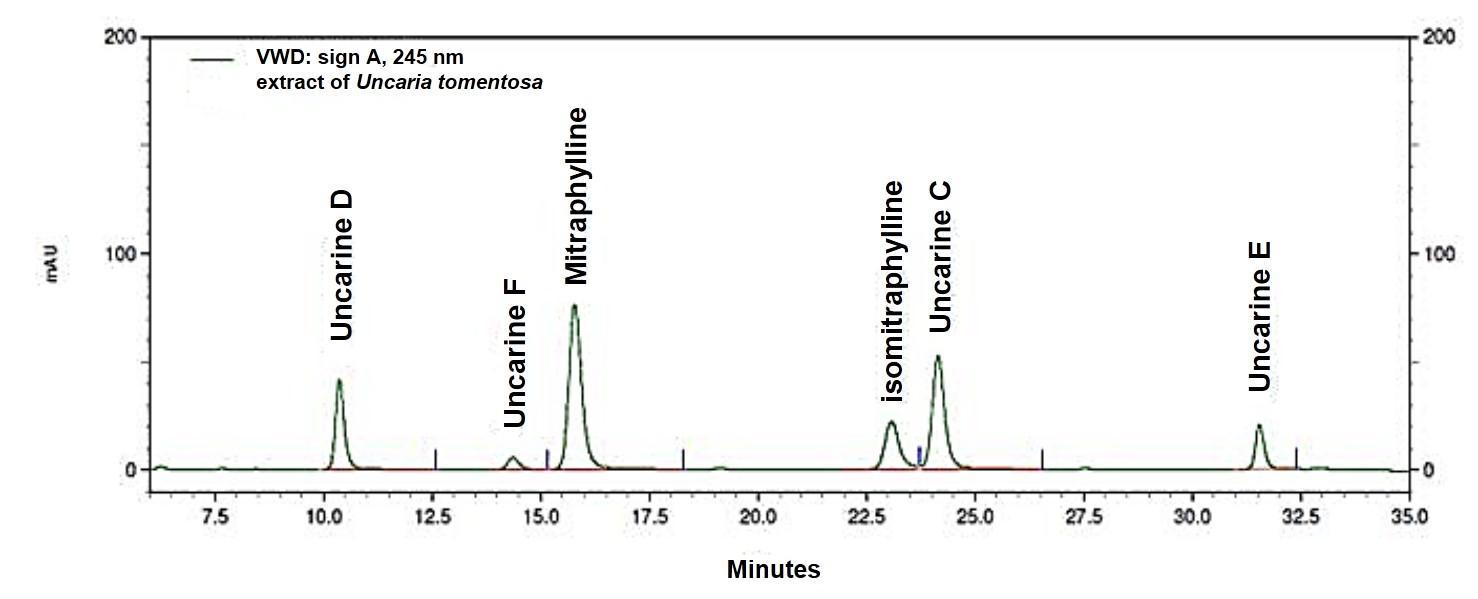


**SI 1:** Chromatogram of *Uncaria tomentosa* crude extract used in the experiments, showing most of the alkaloids such as uncarine D, uncarine F, isomitraphylline, uncarine C, uncarine E, as well as mitraphylline (5.97%), which is a major component of the oxindole alkaloids. The crude extract compounds presented until one hundred milli-Absorbance Units (mAU), for thirty-five minutes, measured by high-performance liquid chromatography (HPLC).
